# HIF-2α upregulation mediated by hypoxia promotes NAFLD-HCC progression by activating lipid synthesis via the PI3K-AKT-mTOR pathway

**DOI:** 10.18632/aging.102488

**Published:** 2019-12-04

**Authors:** Jianxu Chen, Jiandi Chen, Jiaxin Huang, Zhanyu Li, Yihang Gong, Baojia Zou, Xialei Liu, Lei Ding, Peiping Li, Zhiquan Zhu, Baimeng Zhang, Hui Guo, Chaonong Cai, Jian Li

**Affiliations:** 1 Department of Hepatobiliary Surgery, The Fifth Affiliated Hospital of Sun Yat-sen University, Zhuhai, China; 2 Department of Endocrinology, The Fifth Affiliated Hospital of Sun Yat-sen University, Zhuhai, China; 3 Department of Medical Oncology, Cancer Center, West China Hospital, Sichuan University, Chengdu, China; 4 Department of Pathology, The Fifth Affiliated Hospital of Sun Yat-sen University, Zhuhai, China; 5 Department of Thoracic Oncology, The Cancer Center of The Fifth Affiliated Hospital of Sun Yat-sen University, Zhuhai, China; 6 Department of Interventional Radiology, The Fifth Affiliated Hospital of Sun Yat-sen University, Zhuhai, China

**Keywords:** hepatocellular carcinoma, non-alcoholic fatty liver disease, microenvironment, HIF-2α, lipid metabolism

## Abstract

Non-alcoholic fatty liver disease (NAFLD) is a relevant risk factor for developing hepatocellular carcinoma (HCC). Steatohepatitic HCC (SH-HCC), characterized by HCC with steatosis, is influenced by lipid metabolism disorders. A hypoxic microenvironment is common in HCC and affects lipid metabolism. However, whether hypoxia-induced HIF-2α upregulation exacerbates lipid accumulation to contribute to SH-HCC progression remains unclear. In this study, we demonstrated that HIF-2α was elevated in tissues from NAFLD-HCC patients and was associated with survival. Under hypoxic conditions, upregulated HIF-2α was accompanied by lipid accumulation and PI3K-AKT-mTOR pathway activation. HIF-2α knockdown (KD) in steatotic HCC ameliorated triglyceride accumulation and steatosis. HIF-2α-KD steatotic HCC showed minimal lipid synthesis in a hypoxic environment, which contributes to a reduction in malignant behaviours. However, treatment with MHY1485 restored these behaviours. STAM mice, a mouse model that develops NAFLD-HCC, exhibit more rapid tumour progression upon exposure to hypoxia. STAM mice treated with INK-128 presented abrogated mTOR expression and tumour progression under hypoxic conditions with lower triglycerides and steatosis. In conclusion, in a hypoxic microenvironment, HIF-2α upregulation promotes steatotic HCC progression by activating lipid synthesis via the PI3K-AKT-mTOR pathway. Therefore, HIF-2α can be a biomarker and target in developing specific therapeutic measures for NAFLD-HCC patients.

## INTRODUCTION

Liver cancer, a leading cause of cancer-related death, is the sixth most common cancer worldwide [1]. Hepatocellular carcinoma (HCC), a major histological subtype of primary liver cancers, accounts for 70%–85% of cases. Nonalcoholic fatty liver disease (NAFLD) associated with lifestyle changes is the most popular chronic liver disease and has become a relevant risk factor of developing HCC [2]. NAFLD is a dynamic process that involves defective lipid utilization, increased lipid influx into the liver, and de novo hepatic lipogenesis, all of which contribute to hepatic lipid accumulation. Moreover, NAFLD might progress to non-alcoholic steatohepatitis (NASH), cirrhosis, and, finally, HCC. [3, 4]. In clinical practice, HCC is considered the most overlooked outcome of NAFLD. Incomplete knowledge of the underlying mechanisms in carcinogenesis and NAFLD-HCC progression impedes the development of specific therapeutic measures for NAFLD-HCC patients.

Steatohepatitic HCC (SH-HCC), a subcategory of HCC characterized by HCC with steatosis, has been recently recognized as associated with NAFLD [5]. Its features include large droplet steatosis, ballooning of malignant hepatocytes. Although SH-HCC occurs almost exclusively in patients with underlying steatohepatitis, its pathogenesis has yet to be elucidated [6]. Lipid metabolism, including altered fatty acid (FA) metabolism, might play a leading role in developing SH-HCC. Moreover, disrupted FA metabolism is characteristic of tumor cells and promotes tumor progression [7–9]. Most tumor cells, including HCC, synthesize FAs de novo [7, 10]. It has been reported that the HIF-2α (EPAS1) gene participates in FA metabolism [11–14]. Whether HIF-2α influence NAFLD-HCC progression remained unknown and the underlying mechanism is unclear.

Hypoxia is profound in different solid tumours, and its significance has been reviewed before [15]. Energy metabolism driving cellular bioenergetics requires oxygen. Liver mitochondria act as the important regulator of hepatocellular energy metabolism because ATP synthesis and FA oxidation occur in these organelles. Oxygen consumption in mitochondria is considered a factor influencing lipid metabolism during hypoxia. Regions with low oxygen uptake are considered hypoxic regions. The rapid growth of a tumour mass outstrips the process of vascularization, leading to a hypoxic microenvironment in solid tumours [16]. Moreover, such a microenvironment forces tumour to adapt to hypoxia by changing their gene expression patterns to combat hypoxia-induced cell death. A tumour microenvironment with a low O_2_ levels is a contributing factor in increased metastasis rates [17], poorer patient survival [18] and some drug resistance [19].

The hypoxic tumour region induces the overexpression of hypoxia-inducible transcription factor (HIF) genes that contribute to the activation genes related to proliferation, invasion and metastasis [20]. The HIF family plays a crucial role in responding to hypoxic stress. Three isoforms exist in mammals: HIF-1, HIF-2, and HIF-3. Each HIF protein consists of an O_2_-sensitive α subunit (HIF-1α, HIF-2α or HIF-3α) and the O_2_-insensitive HIF-β subunit. In mammals, HIF-α proteins are regulated primarily by three oxygen-dependent prolyl hydroxylase domain (PHD) isoforms (PHD1,2, and 3) among which PHD2 is the most abundant and widely expressed hydroxylase. Under normoxic conditions, HIF-α is a target of the Von Hippel-Lindau tumour suppressor protein pVHL, which catalyses HIF-α degradation via PHD proteins. Under hypoxic conditions, PHDs change to inactive state and HIF-α is no longer hydroxylated. HIF-α subunits, including HIF-2α, translocated to the nucleus to activate the transcription of their target genes by binding to hypoxic response elements [13]. However, most studies focus on the function of HIF-1α in tumour progression and ignore the participation of HIF-2α.

HIF-2α, also known as EPAS1 (Homo sapiens endothelial PAS domain protein 1), influences some biological functions, such as angiogenesis, energy metabolism, cell migration, and tumour invasion. Moreover, HIF-1α mainly regulates glucose metabolism for ATP generation in the absence of oxygen by activating several essential glycolytic enzymes [21]. In addition, it has been reported that HIF-2α promotes steatohepatitis by regulating lipid metabolism [11–14]. Because oxygen consumption in mitochondria significantly affects FA oxidation under hypoxia, it is reasonable to hypothesize that lipid metabolism should be altered via HIF-2α activation induced by hypoxia microenvironment.

In this study, we determined whether hypoxia-induced HIF-2α overexpression regulates lipid metabolism to promote tumour progression in NAFLD-HCC patients. However, the underlying mechanism by which HIF-2α might influence lipid metabolism in steatotic HCC cells in a hypoxic microenvironment has not been definitively determined.

## RESULTS

### HIF-2α, but not HIF-1α, was upregulated in NAFLD-HCC patients

**Figure 1:**
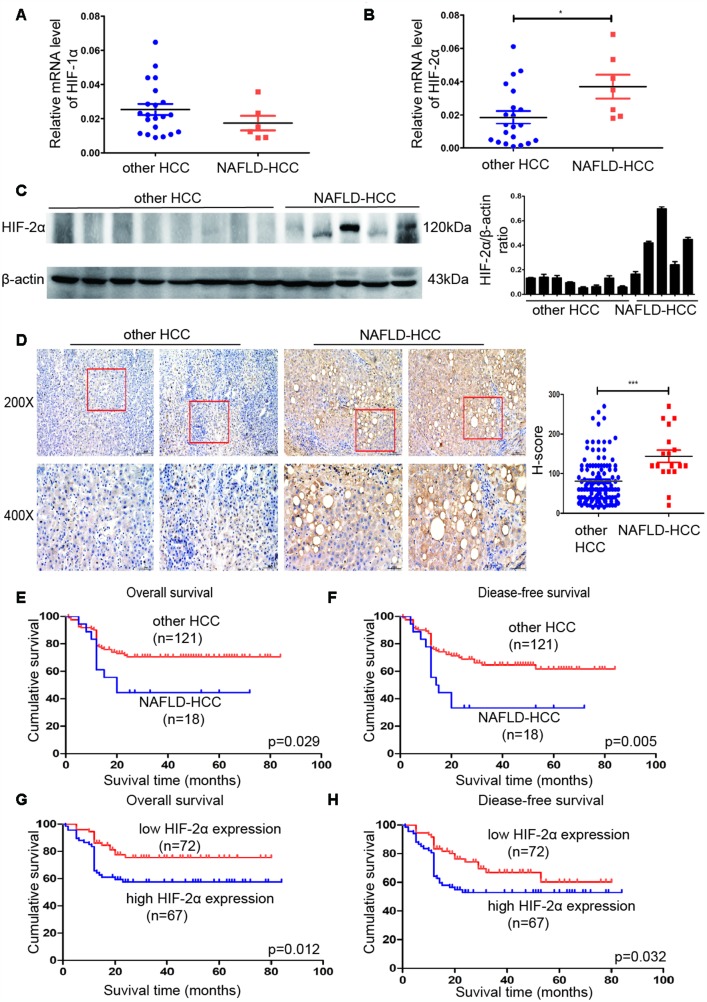
**HIF-2α was upregulated in NAFLD-HCC patients and correlated with poor survival.** (**A**, **B**) Quantitative RT-PCR assessment of HIF-1α and HIF-2α expression in tissues from NAFLD-HCC patients (n=7) and other HCC patients (n=21). Transcription levels were normalized to those of β-actin. (**C**). Western blot analysis of HIF-2α expression in the patients’ tissues. β-Actin was used as the loading control. Densitometric analyses of the band intensity ratios for HIF-2α/β-actin. (**D**) IHC determination of HIF-2α expression in tissues from HCC and NAFLD-HCC patients (magnification: 200X, 400X). H score between NAFLD-HCC and HCC (**E**, **F**) Survival curves of 139 patients with HCC and NAFLD-HCC. (**G**, **H**) Survival curves of 139 patients stratified according to HIF-2α protein expression.

To investigate whether HIFs are involved in NAFLD-HCC, experiments were performed with human HCC tissues. The mRNA levels of HIF-2α (Figure 1B), but not HIF-1α (Figure 1A), were significantly higher in NAFLD-HCC patient tissues than in other HCC tissues. We further analysed HIF-2α protein expression in other HCC and NAFLD-HCC patient tissues by Western blotting (Figure 1C). These results indicated that the mRNA and protein expression levels of HIF-2α were significantly upregulated in NAFLD-HCC patient tissues. The IHC results (Figure 1D) show the same trends. A representative picture showing upregulated HIF-2α mRNA and protein levels in NALFD-HCC patients suggest that HIF-2α may play a role in NAFLD-HCC.

### HIF-2α expression levels in HCC were correlated with clinical progression and poor survival

IHC was used to study the expression of HIF-2α in 139 paraffin-embedded HCC tissues. The cut-off value was set as previously described. The number of cases with high HIF-2α expression was 67, and the remaining cases were deemed low HIF-2α expression. Moreover, to determine the possible relationship between HIF-2α expression and clinicopathologic features in our cohort, we analysed the clinical data by Pearson Chi-squared test (Table 1). As shown in Table 1, high HIF-2α expression was correlated with tumour size (P=0.001), tumour number (P=0.021) and alanine aminotransferase (P<0.001). The correlation between HIF-2α expression and clinical factors was further investigated with univariate and multivariate analyses. As shown in Table 2, high HIF-2α levels were significantly associated with tumour size (95%CI 1.611-8.178), tumour number (95%CI 1.289-22.174), alanine aminotransferase (95%CI 2.775-15.409) and age (95%CI 1.203-6.068).

**Table 1:** Correlation between the clinicopathologic variables and HIF-2α in HCC

**Variables**	**Total**	**HIF-2α expression**		**P value Chi-square**
		**Low expression**	**High expression**	
**Age(years)**				
≤50	74(53.2%)	44(59.5%)	30(40.5%)	0.054
>50	65(48.6%)	28(43.1%)	37(56.9%)	
**Gender**				
male	116(83.5%)	58(50%)	58(50%)	0.341
female	23(16.5%)	14(60.9%)	9(39.1%)	
**TMN** **stage**				
I	107(77.0%)	59(55.1%)	48(44.9%)	0.108
II	9(6.5%)	4(44.4%)	5(55.6%)	
III	5(3.6%)	0(0%)	5(100%)	
IV	18(12.9%)	9(50%)	9(50%)	
**Lymph node metastasis**				
no	122(87.8%)	64(52.5%)	58(47.5%)	0.676
yes	17(12.2%)	8(47.1%)	9(52.9%)	
**Tumor size, cm**				
≤5 cm	85(61.2%)	54(63.5%)	31(36.5%)	0.001^**^
>5 cm	54(38.8%)	18(33.3%)	36(66.7%)	
**Tumor** **number**				
solitary	123(88.5%)	68(55.3%)	55(44.7%)	0.021^*^
multiple	16(11.5%)	4(25%)	12(75%)	
**Tumor recurrence**				
no	128(92.1%)	66(51.6%)	62(48.4%)	0.849
yes	11(7.9%)	6(54.5%)	5(45.5%)	
**Metastasis**				
no	137(98.6%)	70(51.1%)	67(48.9%)	0.169
yes	2(1.4%)	2(100%)	0(0%)	
**ALT**				
normal	57(41%)	42(73.7%)	15(26.3%)	<0.001^***^
increase	82(59%)	30(36.6%)	52(63.4%)	
**Total** **bilirubin**				
normal	88(63.3%)	47(53.1%)	41(46.6%)	0.618
increase	51(36.7%)	25(49%)	26(51%)	
**AFP**				
<400	100(71.9%)	52(52.0%)	48(48%)	0.939
≥400	39(28.1%)	20(51.3%)	19(48.7%)	
**HBsAg**				
negative	36(25.9%)	20(55.6%)	16(44.4%)	0.600
positive	103(74.1%)	52(50.5%)	51(49.5%)	
**Albumin**				
normal	77(55.4%)	43(55.8%)	34(44.2%)	0.287
decrease	62(44.6%)	29(46.8%)	33(53.2%)	

TNM, tumor-node-metastasis; ALT, Alanine aminotransferase; HBsAg, hepatitis B surface antigen; AFP, alpha-fetoprotein

**Table 2:** Univariate and multivariate analyses of clinicopathologic variables and HIF-2α in HCC

**Variables**	**Univariate analysis P value**	**Multivariate analysis 95%CI**	**P value**
Age (≤50/>50y)	0.055	1.203-6.068	0.013^*^
Gender(female/male)	0.343		
TMN stage	0.273		
Lymph node metastasis(yes/no)	0.677		
Tumor size (≤5/>5 cm)	0.001^**^	1.611-8.178	0.001^**^
Tumor numbers (Solitary/Multiple)	0.030^*^	1.289-22.174	0.013^*^
Tumor recurrence (No/Yes)	0.849		
Metastasis (No/Yes)	0.103		
ALT (normal/increase)	<0.001^***^	2.775-15.409	<0.001^***^
Total bilirubin (normal/increase)	0.618		
AFP (<400/≥400)	0.939		
HBsAg (Negative/Positive)	0.600		
Albumin(normal/decrease)	0.288		

CI, confidence interval; TNM, tumor-node-metastasis; ALT, Alanine aminotransferase; HBsAg, hepatitis B surface antigen; AFP, alpha-fetoprotein

We investigated the survival of other HCC and NAFLD-HCC patients (Figure 1E, 1F). NAFLD-HCC patients have a worse survival than other HCC patients, which is consistent with a previous study [22]. We further explored the possible correlation between HIF-2α level and patient survival. We used Kaplan-Meier survival analysis and discovered that patients with increased HIF-2α expression experienced worse outcomes than patients exhibiting low HIF-2α expression (Figure 1G, 1H). The correlation between HIF-2α overexpression and clinical survival in NAFLD-HCC was shown in Supplementary Figure 1D, 1F. The clinicopathological data and survival curves suggest that HIF-2α plays a role in HCC clinical progression and poor survival.

### Hypoxic microenvironment induced lipid accumulation in steatotic HCC cells by upregulating HIF-2α

**Figure 2:**
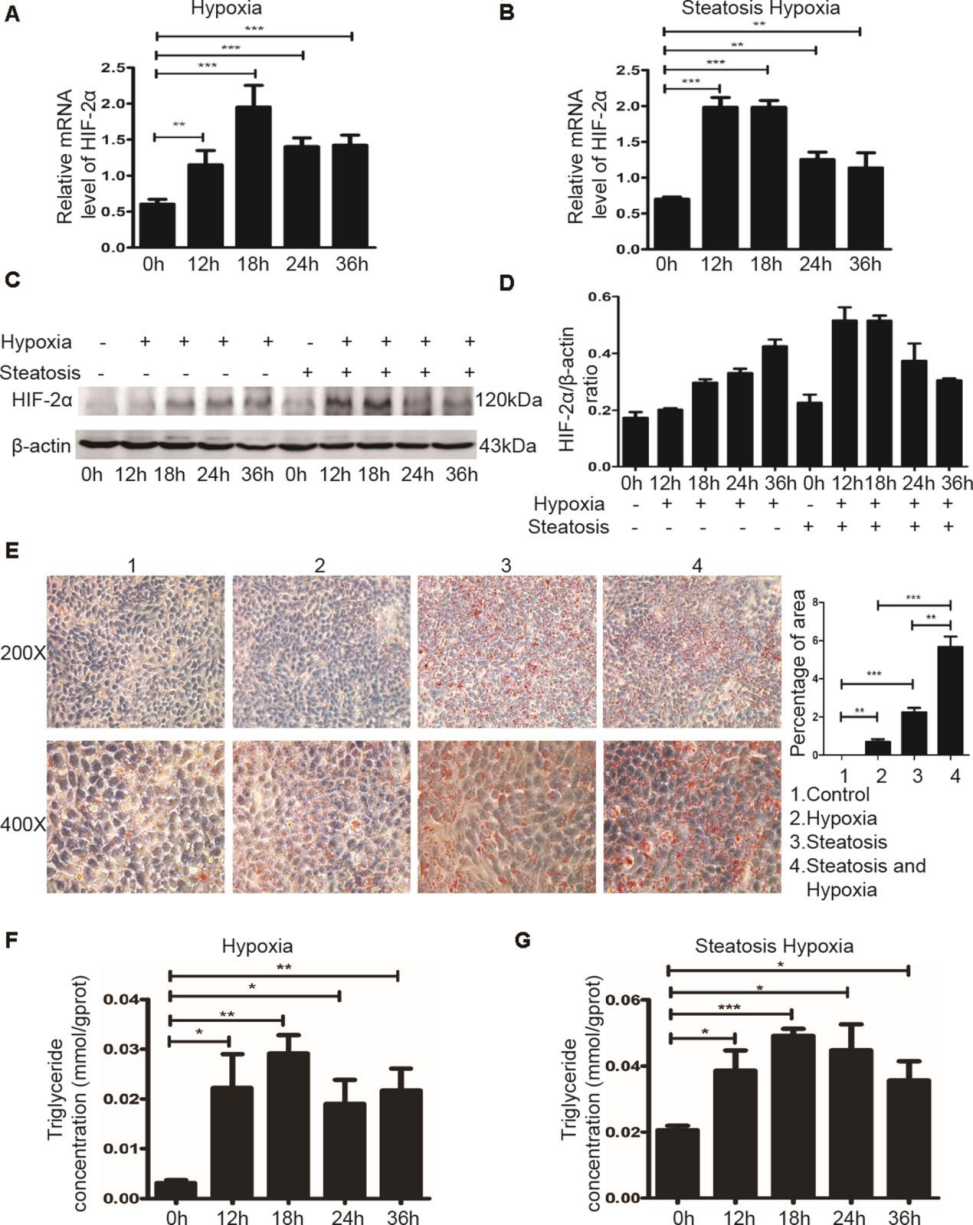
**The hypoxic microenvironment induces lipid accumulation in HCC and steatotic HCC cells by upregulating HIF-2α.** (**A**, **B**) Quantitative RT-PCR assessment of HIF-2α expression in HCC and steatotic HCC cells under hypoxic conditions. Transcription levels were normalized to those of β-actin. (**C**) Western blot analysis of HIF-2α expression in HCC and steatotic HCC cells under hypoxic conditions. β-Actin was used as the loading control. (**D**) Densitometric analyses of the band intensity ratios for HIF-2α/β-actin. (**E**) Oil red O staining and quantification in HCC and steatotic HCC cells with or without hypoxia treatment. (**F**, **G**) Triglyceride levels in HCC and steatotic HCC cells subjected to different durations of hypoxia.

Because a hypoxic microenvironment is common in tumour tissues, we utilized a hypoxia chamber to mimic this microenvironment. To investigate the role of HIF-2α in steatotic HCC, we transferred HCC and steatotic HCC cells to a hypoxia chamber at different times. The mRNA and protein levels of HIF-2α are displayed in Figure 2A–2D. Compared with standard cell culture (control group), cells grown in a hypoxic microenvironment showed increased HIF-2α expression. Moreover, steatotic HCC presented more lipid accumulation in the hypoxic environment, and HCC showed a similar tendency (Figure 2F–2G). We also detected the triglyceride level with Oil red O straining, which followed the same trend (Figure 2E). These results suggest that hypoxia modulated HIF-2α upregulation and promoted lipid accumulation in steatotic HCC.

### Hypoxia-induced HIF-2α expression activated mTOR signalling pathways

**Figure 3:**
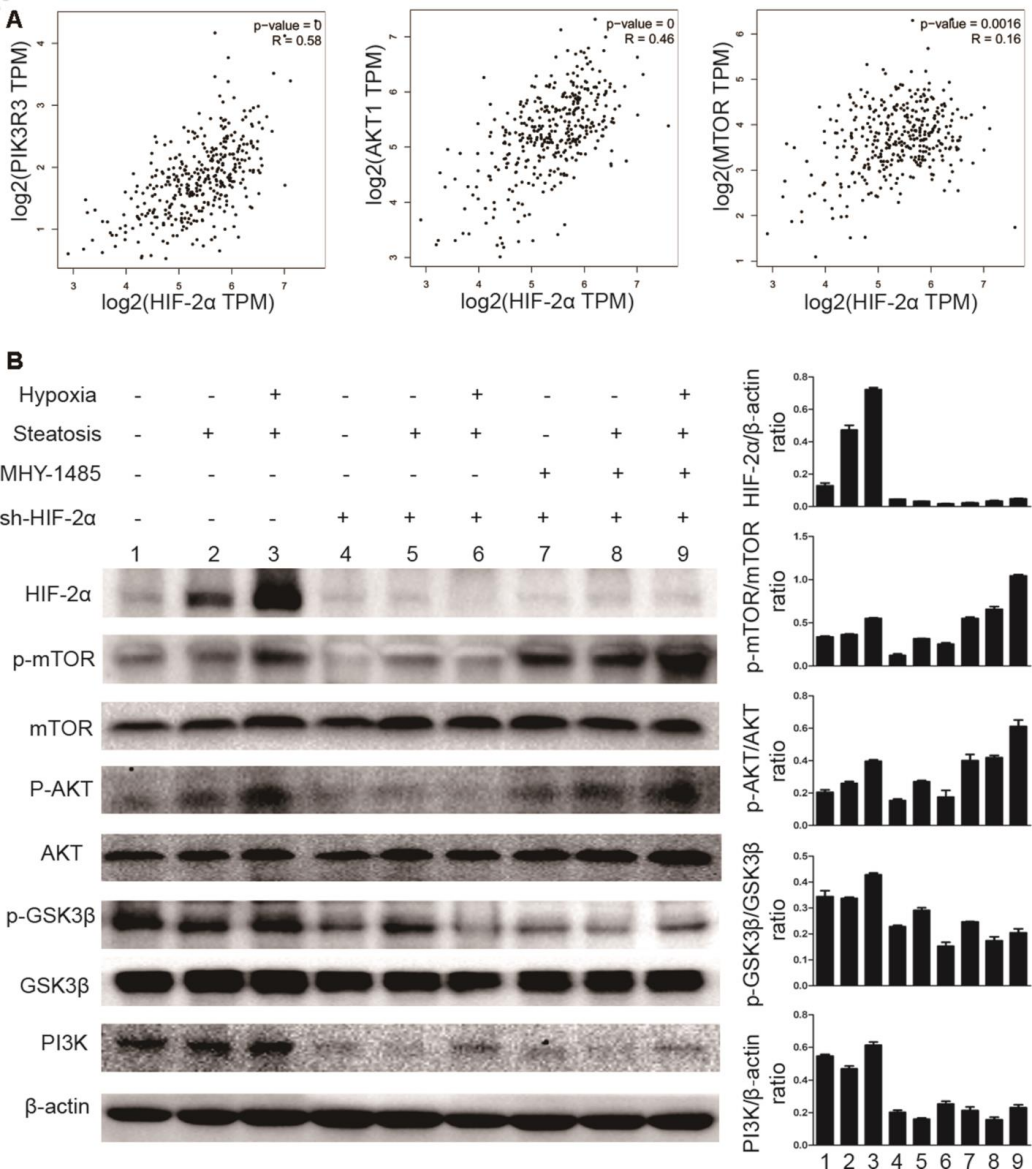
**Hypoxia-mediated upregulation of HIF-2α expression activates mTOR signalling pathways.** (**A**) Pearson correlation analysis of the correlation between HIF-2α expression and relative genes in the patients with liver hepatocellular carcinoma (LIHC) from the TCGA database (http://gepia.cancer-pku.cn). (**B**) Western blot analysis of HIF-2α expression and expression of PI3K-AKT-mTOR pathway factors in HCC and steatotic HCC under hypoxic conditions. β-Actin was used as the loading control. Densitometric analyses of the band intensity ratios for HIF-2α/β-actin, PI3K/β-actin, p-GSK3β/GSK3β, p-AKT/AKT and p-mTOR/mTOR.

To study the underlying mechanism, we downloaded the list of genes correlated with HIF-2α in HCC from The Cancer Genome Atlas (TCGA) database (http://gepia.cancer-pku.cn) (Supplementary Table 1). These data suggested that HIF-2α is associated with PI3K, AKT and mTOR (Figure 3A).

Because the PI3K-AKT-mTOR pathway has been shown to control tumour development and lipid synthesis, we tested whether HIF-2α affects PI3K-AKT-mTOR signalling in steatotic HCC cells. We also detected the expression of PI3K, AKT, mTOR, GSK3B and the phosphorylation level of AKT at Ser473, mTOR at S2448, and GSK3B at Ser9 in steatotic HCC cells (Figure 3B). When cells were treated with MHY1485, an mTOR selective agonist, p-mTOR was upregulated, proving its effectiveness. Under normoxic conditions, HIF-2α expression was also elevated in steatotic HCC compared with that in HCC. We found that, in hypoxic conditions, HIF-2α overexpression is accompanied by increases in p-AKT, p-GSK3B and p-mTOR. After transfection with sh-HIF2α, the levels of PI3K, p-AKT, p-mTOR and p-GSK3B decreased significantly. This result may indicate that hypoxia upregulates HIF-2α expression, activating PI3K-AKT-mTOR signalling.

### Steatotic HCC growth, migration, invasion and angiogenesis can be ameliorated by intervention of the HIF-2α/PI3K-AKT-mTOR signalling axis

To further investigate the effect of HIF-2α on tumour biological function, we inhibited HIF-2α expression by transfecting cells with sh-HIF2α. Western blotting and quantitative RT-PCR determined that sh-HIF2α effectively downregulated HIF-2α expression (Supplementary Figure 1B, 1C).

**Figure 4:**
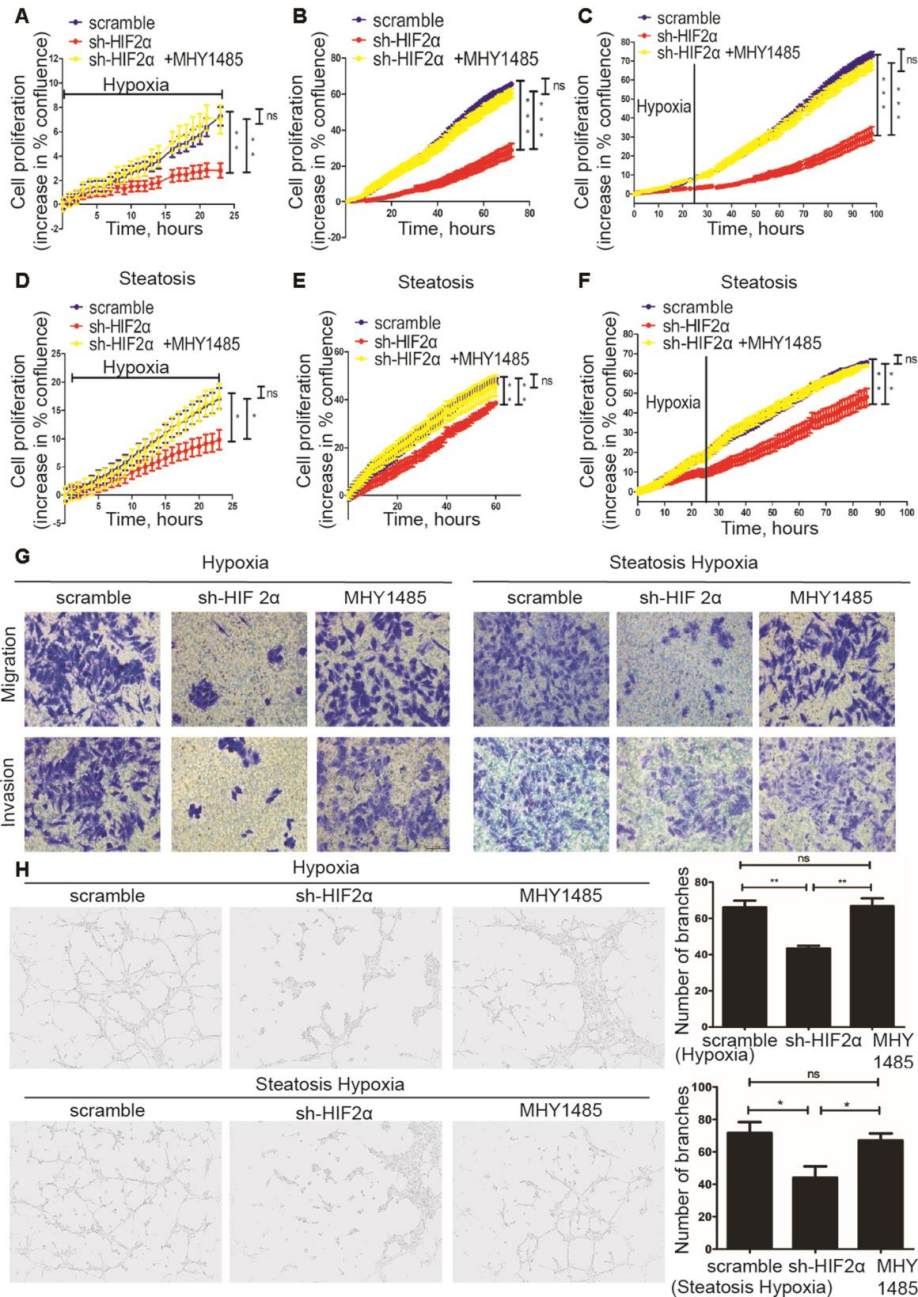
**Steatotic HCC growth, migration, invasion and angiogenesis in a hypoxic microenvironment needs HIF-2α.** (**A**–**C**) Summary graph showing the HCC cell 2D growth rate in hypoxic conditions (**A**), after 24 hypoxia treatments (**B**) and all conditions (**C**). (**D**–**F**) Summary graph showing the steatotic HCC cell 2D growth rate under hypoxic conditions (**D**) after 24 hypoxia treatments (**E**) and all conditions (**F**, **G**). Transwell assays of HCC, HIF-2α-KD HCC, steatotic HCC and HIF-2α-KD steatotic HCC cells under hypoxic conditions in the presence or absence of MHY1485 treatment. h. Representative images of HUVEC tube formation; HUVECs were treated with the supernatant of HCC, HIF-2α-KD HCC, steatotic HCC and HIF-2α-KD steatotic HCC cell cultures.

For 2D growth assays, a live real-time IncuCyte was utilized. In response to hypoxia treatment, HIF-2α-KD cells exhibited less survival in low O_2_ concentrations and presented a relatively lower proliferation rate than did the scramble group (Figure 4A). After 24 h of hypoxia treatment, the cells were transferred to a normoxia environment. HIF-2α knockdown also suppressed the growth of HCC under normal oxygen conditions after hypoxia treatment (Figure 4B). With regard to the overall growth curve, HIF-2α-KD cells had less growth than cells transfected with a control lentivirus (Figure 4C). The same trend could be found in steatotic HCC (Figure 4D–4F). For the transwell assay, HIF-2α-KD cells reduced tumour cell migration and invasion (Figure 4G). The in vitro tube formation assay showed more vessels in the scrambled group than in the HIF-2α-KD group (Figure 4H, P<0.005). We also activated p-mTOR with MHY1485, a mTOR-selective agonist, to study the role of the mTOR pathway in this process. In the HIF-2α-KD group, the effect of sh-HIF2α was reversed after MHY1485 treatment. Moreover, steatotic HCC showed the same tendency. Transwell assays and tube formation were all performed in a hypoxic environment. The results of the functional assays suggest that HIF-2α promotes growth, migration, invasion and angiogenesis of HCC and steatotic HCC under hypoxic conditions, and these effects can be rescued by MHY1485. Furthermore, HIF-2α influenced the biological function of HCC via the PI3K-AKT-mTOR pathway.

### HIF-2α induced lipid synthesis and exacerbated lipid accumulation in steatotic HCC cells under a hypoxic microenvironment

As HIF-2α can regulate cell biological function under hypoxic conditions and mTOR complexity may modulate cell metabolism, we further studied the effect of HIF-2α on lipid synthesis.

**Figure 5:**
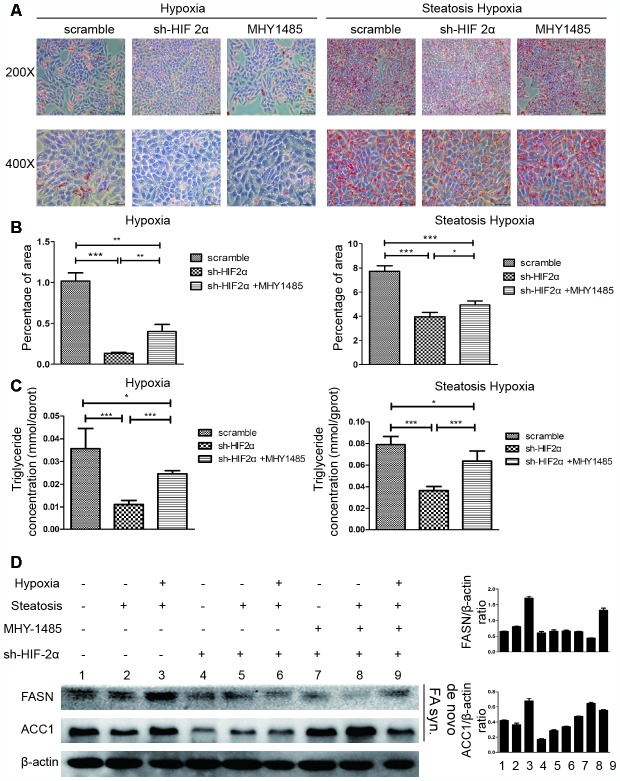
HIF-2α induces steatotic HCC lipid synthesis under a hypoxic microenvironment (**A**) Oil red O staining and quantification in HCC and steatotic HCC under hypoxic conditions in the presence or absence of MHY1485. (**B**, **C**) Triglyceride levels in HCC and steatotic HCC under hypoxic conditions in the presence or absence of MHY1485. (**D**) Western blot analysis of lipid synthesis-related proteins in HCC and steatotic HCC under hypoxic conditions. Densitometric analyses of the band intensity ratios for ACC1/β-actin and FASN/β-actin.

Upon hypoxia treatment, HIF-2α-KD cells exhibited ameliorated triglyceride accumulation and steatosis compared to cells in the scramble group, and the effect was reversed by MHY1485 (Figure 5A–5C). To assess the link between HIF-2α upregulation and lipid synthesis, we employed Western blotting to evaluate the level of lipid synthesis-related proteins, such as FASN and ACC1 (Figure 5D). The results demonstrated that increases in HIF-2α expression are accompanied with the upregulation of lipid synthesis-related protein in response to hypoxia. Moreover, cells transfected with sh-HIF2α showed dramatically decreased FASN and ACC1 expression. When cells were treated with MHY1485, FASN and ACC1 was rescued and elevated to the previous endogenous levels. These results suggest that HIF-2α promotes lipid synthesis in steatotic HCC under hypoxic conditions.

### Hypoxia-induced HIF-2α overexpression promoted tumour development and lipid accumulation in STAM mice in vivo, which can be rescued by an mTOR inhibitor

STAM mice, a mouse model characterized by its resemblance to consistent progression to NALFD-HCC in humans, were treated to induce diabetes and fed a high-fat diet. To investigate the long-term effect of hypoxia-induced HIF-2α, we mimicked the hypoxia environment in the cages and treated mice with INK-128.

**Figure 6:**
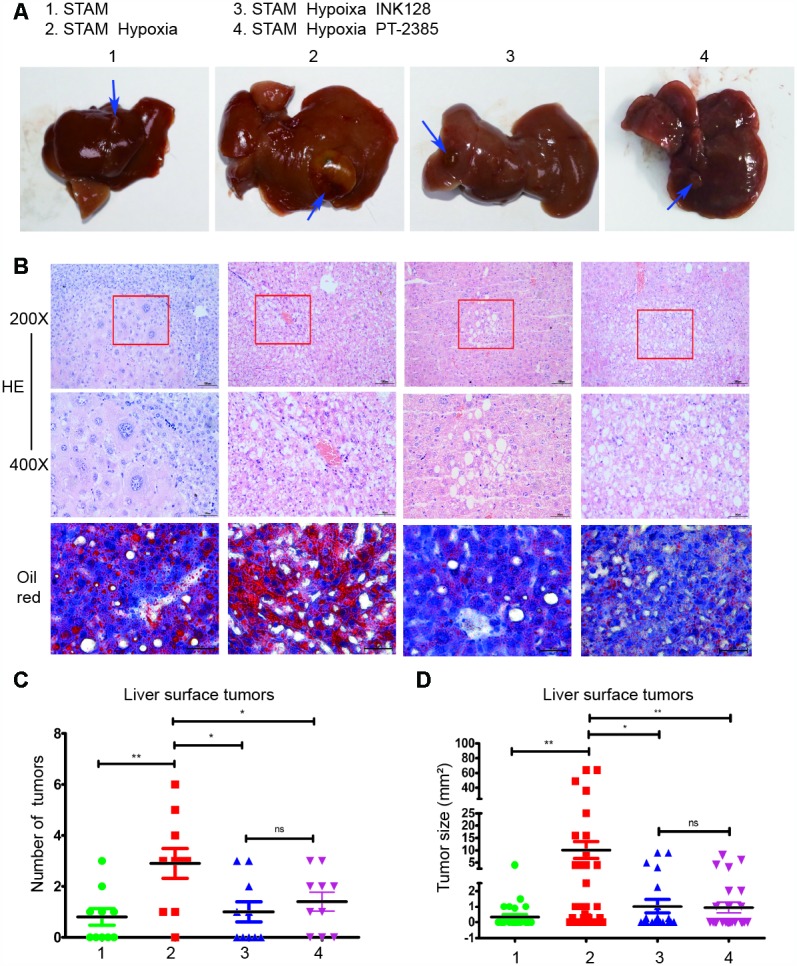
**Hypoxia-induced HIF-2α overexpression promotes STAM mouse tumour development and lipid accumulation in vivo, which can be rescued by an mTOR inhibitor.** (**A**). Gross specimens from STAM mice of different groups. (**B**) Images of liver stained by HE and Oil red O staining. (**C**) The numbers of tumours on the liver surface of different STAM mice (n = 10 mice/group). d. In the tumour size analysis, the largest three tumours found at a random location in the liver were used.

When exposed to a hypoxic environment, STAM mice developed 2.9±1.9 surface tumours compared to 0.8±1.0 tumours in the untreated STAM group (p=0.005) (Figure 6C). After INK-128 treatment during the hypoxia intervention, the number of surface tumours decreased to 1.1±1.3 compared with the hypoxia group (p=0.015) (Figure 6C). Moreover, after PT-2385 treatment, STAM mice developed less tumours (1.4±1.2) compared with the hypoxia group. Similar to hypoxia treatment, tumour sizes were significantly increased in hypoxic STAM mice compared with untreated STAM mice (p=0.005) (Figure 6D). Tumours in the INK-128-treated group were also significantly smaller (p=0.011) (Figure 6D). Tumour sizes were decreased in PT-2385 group compared with Hypoxia STAM mice. As shown in Figure 6B, hypoxia STAM mice represent more tumour cell atypia and lipid accumulation than untreated STAM mice. HE staining display pathological changes in different mice group. The steatotic cells and the tumors happened in mice. Moreover, these behaviours can be rescued by INK-128 and PT-2385.

### Hypoxia-induced HIF-2α upregulation altered NAFLD-HCC lipid accumulation via the PI3K-mTOR pathway in vivo

**Figure 7:**
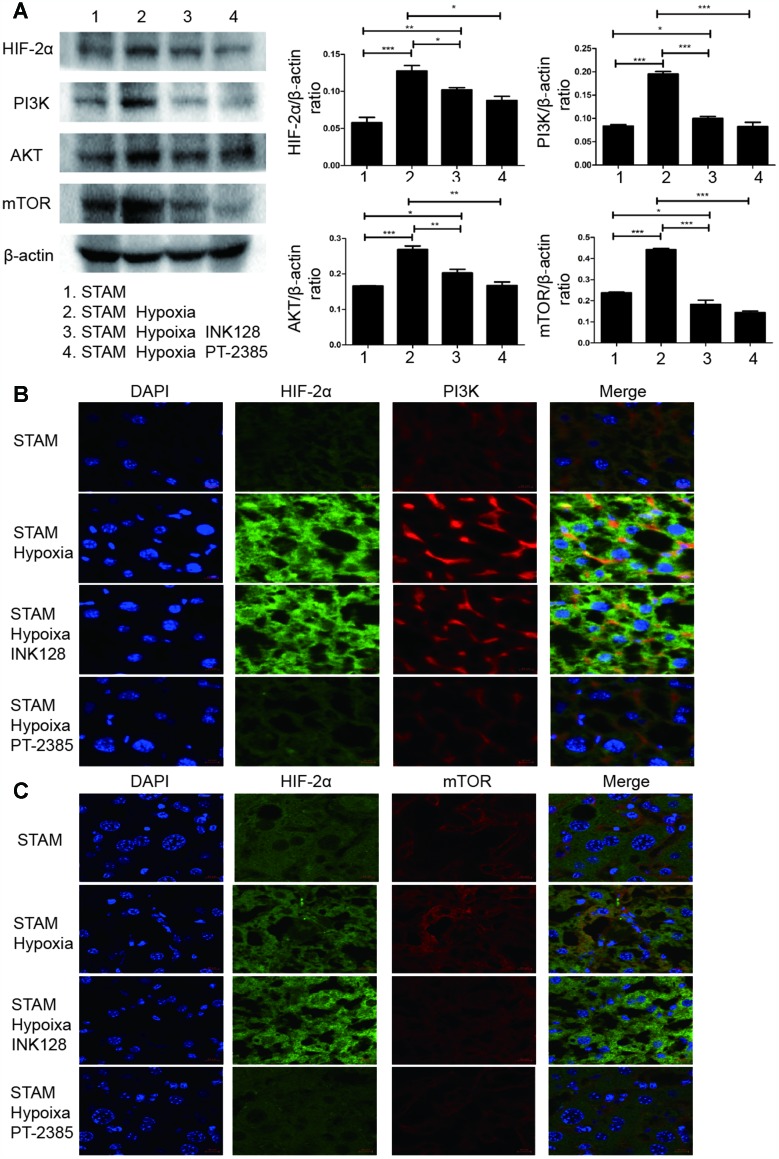
**Hypoxia-induced HIF-2α overexpression promotes STAM mouse tumour development via activating the PI3K-AKT-mTOR signalling axis.** (**A**) Western blot analysis of HIF-2α expression and expression of PI3K-mTOR pathway factors in STAM mice of different groups. β-Actin was used as the loading control. Densitometric analyses of the band intensity ratios for HIF-2α/β-actin, PI3K/β-actin and mTOR/β-actin. (**B**, **C**) The liver sections were stained with DAPI to visualise nuclei (blue), Alexa Fluor 488 to visualise the distribution of HIF-2α (green) and Alexa Fluor 568 to PI3K/mTOR (red) proteins. Scale bar: 10 μm.

To determine the underlying mechanism of how HIF-2α manipulates NAFLD-HCC lipid accumulation, we detected HIF-2α, PI3K and mTOR expression by Western blotting. After hypoxia intervention, HIF-2α was upregulated, accompanied by PI3K and mTOR elevation (Figure 7A). Moreover, with INK-128 treatment, STAM mice suppressed mTOR expression and lipid accumulation, which contribute to restraining the progression of NAFLD-HCC.

**Figure 8:**
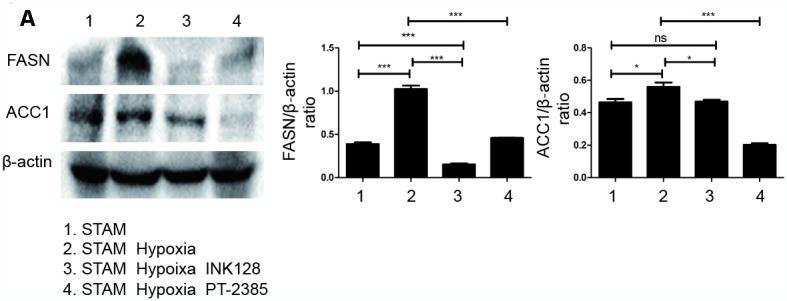
**HIF-2α induced lipid synthesis and exacerbated lipid accumulation in STAM mice in vivo.** (**A**), Western blot analysis of FASN and ACC1 expression in STAM mice of different groups. β-actin was used as the loading control. Densitometric analyses of the band intensity ratios for FASN/β-actin and ACC1/β-actin.

We further investigated HIF-2α, PI3K, mTOR and Akt expression and distribution in STAM mouse tumour frozen sections. HIF-2α expression was significantly elevated with hypoxia intervention and shown to colocalize with PI3K by confocal microscopy (Figure 7B). With INK-128 treatment, mTOR expression was suppressed (Figure 7C). These results suggested that hypoxia-induced HIF-2α upregulation participated in altering NAFLD-HCC lipid accumulation via the PI3K-mTOR pathway. Moreover, we also explore de novo fatty acid synthesis level in mice liver tissues (Figure 8A). With INK-128 or PT-2385 treatment, de novo fatty acid synthesis was suppressed significantly. Therefore, HIF-2α could be a biomarker and target in developing specific therapeutic measures for NAFLD-HCC patients. INK-128 (mTOR inhibitor) and PT-2385 (HIF-2α inhibitor) have a definite role in suppressing NAFLD-HCC progression.

## DISCUSSION

Non-alcoholic fatty liver disease (NAFLD) is a major cause of chronic liver disease worldwide and has become a growing public health problem [23–25]. NAFLD is a continuum of diseases, including steatosis (lipid accumulation) and non-alcoholic steatohepatitis (NASH), and can eventually progress to hepatocellular carcinoma (HCC). NAFLD-HCC also correlates with worse survival, more advanced tumour stage and a lower rate of liver transplantation [23]. NAFLD-HCC stands as the most overlooked outcome and presents different biological characteristics. Steatohepatitic HCC (SH-HCC) is a subcategory of HCC characterized by HCC cells with steatosis and is associated with altered metabolism in obesity-driven HCC, including NAFLD [5, 26]. It is essential to study the mechanism of steatotic HCC to develop specific therapeutic measures for NAFLD-HCC.

Elevated liver lipogenesis is an important contributing factor in the development and progression of NAFLD [27]. Lipid metabolism disorders, including those that increase lipid synthesis, play a part in tumorigenesis and tumour progression [28]. Increased liver lipid synthesis contributes to steatosis, which may correlate with steatohepatitis [29]. However, whether elevated lipid synthesis participates in steatotic HCC cell progression is unclear.

A hypoxic microenvironment is common in various solid tumours. Hypoxia suppresses the immune response and promotes tumour invasiveness, metastasis and drug resistance [30]. To resist hypoxia-mediated tissue necrosis and cell death, tumours alter their gene expression to adapt such conditions. HIF-2α, a member of the hypoxia-inducible factor (HIF) family, is stabilized and translocated to the nucleus under hypoxic conditions. It has been reported that HIF-2α (EPAS1) may promote steatohepatitis via lipid metabolism in normoxic conditions [11–14]. However, the hypoxic environment is profound in solid tumours [15], and the underlying mechanism of how hypoxia-induced HIF-2α upregulation regulates lipid metabolism in steatotic HCC remains unclear. Moreover, the role of HIF-2α under hypoxic conditions in steatotic HCC progression needs to be explored.

Clinically, we investigated HIF-2α and HIF-1α expression levels in NAFLD-HCC patients. The results demonstrate that HIF-2α, but not HIF-1α, was upregulated in tissues from NAFLD-HCC patients (Figure 1A–1D). We further studied the relationship between the expression level of HIF-2α and some clinical characteristics. Tumour size, tumour number and alanine aminotransferase were correlated with HIF-2α expression (Table 1 and Table 2). There are some reasons to explain the correlation. On the one hand, HIF-2α overexpression was induced by the hypoxic microenvironment. Tumour size and tumour number affect hypoxic conditions in a solid tumour due to limited blood supply, which also influences HIF-2α activity and liver function. On the other hand, HIF-2α was also elevated in NAFLD-HCC, which impaired liver function at the NAFLD stage and led to worse liver function. Moreover, high levels of HIF-2α are also associated with worse survival (Figure 1G–1H). These results suggest that HIF-2α may contribute to NAFLD-HCC progression.

To investigate the underlying mechanism, we utilized a steatotic HCC model according to a previous study [31–34]. We also mimicked the hypoxic microenvironment with a hypoxia chamber to study the role of HIF-2α in steatotic HCC progression. Although HIF-2α is relatively upregulated in steatotic HCC, in the hypoxic microenvironment, HIF-2α expression was significantly increased compared with that steatotic HCC cells under normoxic conditions (Figure 2A–2D). Under hypoxic conditions, steatotic HCC cells exhibited more triglyceride and lipid accumulation than under normal oxygen conditions; this accumulation was accompanied by HIF-2α overexpression (Figure 2E–2G). In brief, hypoxia-induced HIF-2α upregulation might be involved in the process of cell steatosis.

We further investigated the molecular mechanism of this increased lipid content. The list of genes correlated with HIF-2α in HCC was downloaded from The Cancer Genome Atlas (TCGA) database (Supplementary Table 1). The data suggested that HIF-2α interacts with the PI3K-AKT-mTOR pathway (Figure 3A), which is considered an essential signal transduction pathway controlling cell growth. A previous study reported that this pathway is an essential signalling pathway that demonstrates elevated activation in tumours [35]. In addition, mTOR, which is part of two functionally distinct protein complexes (mTORC1 and mTORC2), controls metabolism pathways that regulate cell growth [36]. mTORC1, a potential therapeutic target for metabolic disorders, promotes lipid biogenesis by activating the SRPK2 gene, a regulator of RNA-binding SR protein [37]. mTORC2 controls hepatic FA synthesis via AKT and GSK3B and can be a potential target for NAFLD or HCC [28]. Both mTORC1 and mTORC2 are reported as oncogenic proteins that promote tumorigenesis and tumour progression [38–40]. mTOR also modulates the switch to autophagy to regulate HCC growth and proliferation [41]. Therefore, we further studied the interaction between HIF-2α and the PI3K-AKT-mTOR pathway.

Under hypoxic conditions, HIF-2α was upregulated in steatotic HCC, resulting in activated PI3K and increased levels of phosphorylated AKT, GSK3B and mTOR (Figure 3B). We also knocked down HIF-2α by transfecting cells with sh-HIF2α, and its expression is displayed in Supplementary Figure 1B, 1C. The PI3K-AKT-mTOR pathway was inhibited after HIF-2α knockdown (Figure 3B). MHY1485, a mTOR selective agonist, was utilized to activate mTOR for the following experiments. As shown in Figure 3B, hypoxia-induced HIF-2α upregulation activated the PI3K-AKT-mTOR pathway, suggesting that HIF-2α may influence biological cell function via the PI3K-AKT-mTOR pathway.

In the growth assays, transwell assays and tube formation experiments, steatotic HCC exhibited relatively more malignant behaviours than HIF-2α knockdown cells under hypoxic conditions. When treated with MHY1485, HIF-2α-KD cells showed recovered activity similar to that of untreated cells. Moreover, HCC cells show the same tendency (Figure 4A–4H). To investigate lipid accumulation in cells, we studied the triglyceride levels of cells and stained cells with Oil red O (Figure 5A–5C). The triglyceride level and Oil red positive area in cells were decreased after transfection with sh-HIF2α. Protein expression of the lipid synthesis-related genes FASN and ACC1 was explored by Western blot (Figure 5D), the results of which showed that FASN and ACC1 were significantly suppressed when HIF-2α was knocked down. When HIF-2α was upregulated in hypoxic conditions, the expression of lipid synthesis-related protein was elevated. Collectively, in the hypoxic microenvironment, lipid synthesis was elevated by upregulating HIF-2α expression levels.

The underlying mechanism by which mTOR complexes promote tumorigenesis via lipid synthesis has been previously reported [28, 37]. First, mTORC1 has been reported to stimulate FA synthesis in breast cancer cell lines by activating sterol regulatory element-binding proteins [42, 43]. Second, mTORC2 controls the expression of some critical lipid synthesis genes, detailing the mechanism at the transcriptional level [28]. Moreover, the upregulation of lipid synthesis is essential for tumorigenesis and progression. The enhanced lipid synthesis leads to pathological accumulation of FAs, which in turn promote inflammation leading to tumour progression [44]. In this study, we found that in a hypoxic microenvironment, HIF-2α overexpression increases PI3K-AKT-mTOR pathway activity, while mTOR complexes promote steatotic HCC progression via lipid synthesis. Furthermore, hypoxia-induced HIF-2α expression exacerbated lipid accumulation, leading to growth, migration, invasion and angiogenesis in steatotic HCC, which is closely related to NAFLD-HCC.

We also performed experiments in vivo. STAM mice are a mouse model characterized by their resemblance to progression to NALFD-HCC in humans. Hypoxia STAM mouse model was establish to study how HIF-2α manipulates NAFLD-HCC lipid accumulation and promote NAFLD-HCC progression. Our result showed that HIF-2α was significantly upregulated in STAM hypoxia group compare with STAM group. Moreover, in these hypoxic mouse model, liver micro-environment was changed as previously reported [45]. It mimics the tumour micro-environment to some extent. Upon hypoxia exposure, STAM mice develop more tumours than untreated STAM mice. Larger tumours were also found in the hypoxia group. When STAM mice treated with INK-128 (an mTOR inhibitor) were exposed to hypoxia, they developed fewer tumours and smaller tumour sizes. We also performed HE and Oil red O staining on the liver sections. Hypoxia STAM mice showed more tumour cell atypia and lipid accumulation consistent with that observed upon HIF-2α overexpression. As the immunofluorescence images show, the HIF-2α gene colocalized with PI3K, indicating an underlying interaction. These results suggest that hypoxia-induced HIF-2α overexpression promotes NAFLD-HCC progression by activating lipid synthesis via the PI3K-AKT-mTOR pathway.

Steatotic HCC is different from HCC in that it is associated with lipid metabolism disorders and contributes to more malignant behaviours. Under a hypoxic microenvironment, HIF-2α overexpression exacerbates lipid accumulation, leading to steatotic HCC progression via activation of the PI3K-AKT-mTOR pathway. By blocking this signal pathway using a HIF-2α/mTOR agonist, the malignant behaviours were rescued. Therefore, HIF-2α may be the target in developing specific therapeutic measures for NAFLD-HCC.

## METHODS

### HCC specimens from patients

Paraffin-embedded specimens from 139 patients diagnosed with HCC who underwent hepatectomy at the Fifth Affiliated Hospital, Sun Yat-Sen University, China between 2009 and 2015 were investigated. All patient diagnoses were histologically and clinically confirmed. NAFLD-HCC was further proven by an experienced pathologist who evaluated the biopsy samples. Fresh HCC tissues (n=21) and NAFLD-HCC tissues (n=7) were received from the same hospital and stored in liquid nitrogen for further use. The Correlations between the clinicopathologic variables and HIF-2α expression in HCC are presented in Table 1. For the sake of using these patients’ specimens for experimental purposes, all specimens were obtained with informed consent, and the study was approved by the Institutional Ethical Review Board of the Fifth Affiliated Hospital of Sun Yat-Sen University. Moreover, all patients approved the use of their specimens in the following experiments and the analysis of their clinicopathological data.

### Cell lines and cell culture

The human HCC cell line HepG2 was purchased from the Chinese Academy of Sciences, Shanghai Branch (Shanghai, China). HCC cells were cultured in Dulbecco’s modified Eagle’s medium (DMEM; Gibco) supplemented with 10% foetal bovine serum (FBS; Gibco), 50 U/mol penicillin and 50 mg/ml streptomycin (Sigma-Aldrich, USA) and maintained at 37°C with 5% CO_2_.

To investigate SH-HCC, HepG2 cells were treated with free fatty acids (FFAs) (oleate/palmitate, 2:1 ratio); oleate and palmitate (Sigma-Aldrich) were diluted to different concentrations in DMEM containing 10% FBS [31–34]. Cells were serum-starved for 24 h and then treated with different concentrations of FFAs. We chose 1 mmol/L FFAs as the best concentration to establish SH-HCC (Supplementary Figure 1A). To mimic the hypoxia microenvironment, cells were transferred to a hypoxia chamber containing 1% O_2_ [46]. MHY1485, a selective agonist, was utilized to activate the mTOR complex according to the manufacturer’s suggestion.

### Establishment of knockdown HCC cells

A lentiviral-mediated shRNA approach was used to generate knockdown HCC cells. Lentiviral particles expressing either shRNA targeting HIF-2α or a control sequence were constructed by Shanghai Genechem. Lentiviral vectors were used to infect HepG2 cells. After infection, cells were selected with puromycin (Sigma). HIF-2α expression under hypoxic conditions was confirmed by qPCR and Western blotting (Supplementary Figure 1B, 1C).

### Animals and animal models

Pregnant female C57BL/6J mice (180-250 days old) were purchased from the Laboratory Animal Centre of Guangzhou University of Chinese Medicine (Guangzhou, China) and used to establish a model of STAM in which mice are treated to induce diabetes and fed a high-fat diet [47, 48]. In brief, pups birthed within 5 days were injected with 200 μg of streptozotocin (Sigma-Aldrich) and fed with a diet with 60% fat content (D12492; Research Diets). To mimic hypoxia, mice were exposed to 60-s IH cycles (30 s at 5% fraction of inspired oxygen and 30 s at 21% FIO2) for 8 h in their cages between 6 A.M. and 2 P.M. as described in a previous study [45]. Animal experimental protocols were approved by the Animal Ethics Committee of Forevergen Company and performed in the line with the National Institutes of Health guidelines.

### RNA extraction and quantitation and quantitative RT-PCR

Total RNA was extracted from the cultured cells and freshly resected HCC tissues using TRIzol (Life Technologies) according to the manufacturer’s instructions. cDNA was synthesized from total RNA using a RevertAid RT Reverse Transcription kit (K1691). To explore the relative expression levels of different genes, SYBR Green PCR kits (TAKARA) were utilized with the β-actin gene as an internal reference. PCR was performed using a T100™ Thermal Cycler (Bio-Rad) instrument. To ensure the repeatability and authenticity of the results, the experiments were performed in triplicate. Sequences of primers are as follows: β-actin: forward 5′CATCTG CTGGAAGGTGGACA3′, reverse 5′CGACAGGATG CAGAAGGAGA3′; HIF-2α: forward 5′GAGCAAGGA GACGGAGGTGT3′, reverse 5′TGTGTTCGCAGGA AGCTGAT3′.

### Western blotting and immunohistochemistry (IHC)

Protein from cells and tissues was extracted according to a protocol recommended by the manufacturer (P00013C, Beyotime), and the protein concentration was further determined with a BCA kit (Beyotime). For separation, 20 μg of protein was loaded. Primary antibodies targeting HIF-2α (7096), PI3K (4249), GSK-3B (4337), p-GSK3B (5558), AKT (9272), p-AKT (4060), mTOR (2972), p-mTOR (2971), FASN (3180), and ACC1 (4190) were purchased from Cell Signalling Technology. β-Actin (Abcam; ab6276; 1:5000) was used as a loading control. Quantitative analysis of the Western blot data was done using Image J software.

For the IHC assay, the tissues were incubated with HIF-2α at a 1:300 dilution at 4°C for 8 h. For negative controls, the primary antibody was replaced by normal goat serum. Two independent and experienced pathologists who were blinded to the biochemical and clinical patient information scored the immunohistochemically stained tissue. The scoring method was performed as described [49, 50]. For the purposes of quantification and comparison, specimens were assigned an H-score, which multiplies the score corresponding to the percentage of tumour cells stained by the score corresponding to the strength of staining (from 0 for negative staining to 3 for positive staining). The median value in 139 patients served as a cut-off to categorize all cases as high or low HIF-2α expression.

### Immunofluorescence

Immunofluorescence was utilized to study the distribution and expression levels of HIF-2α, PI3K and mTOR in HCC tissues from STAM mice from three groups. We blocked tissues with 5% bovine serum albumin for 60 minutes and incubated them with primary antibodies against HIF-2α (1;200, Cell Signalling Technology), PI3K (1:200, Cell Signalling Technology) or mTOR (1:200, Cell Signalling Technology) overnight at 4°C. PBS was used to rinse the sections 3 times, which were then incubated with Alexa Fluor®568-conjugated goat anti-mouse IgG(H+L) (1:500, Thermo) or Alexa Fluor® 488-conjugated goat anti-rabbit IgG(H+L) (1:500, Thermo) antibodies for one hour at room temperature. DAPI was used to stain cell nuclei. The sections were visualized on a laser confocal microscope (Zeiss).

### Metabolism characteristic assay

A triglyceride assay kit was purchased from Jiancheng Biology Institution PeproTech (Nanjing, Jiangsu, China). Oil red O staining was employed according to a protocol recommended by the manufacturer (Solarbio, G1262). The experiments were performed at least in triplicate.

### Cell proliferation and transwell assay

To measure cell proliferation, a live real-time IncuCyte ZOOM was used as described [51, 52]. For the migration and invasion studies, HepG2 cells or steatotic HepG2 cells were suspended in 100 μl of serum-free medium and placed in a transwell chamber with or without Matrigel. After the cells were incubated at 37°C for 24 h, those attached to the lower surface of the chamber were fixed, stained with crystal violet and counted at a magnification of × 200 in eight randomly selected fields. The experiments were performed at least in triplicate.

### Tube formation assay

Growth factor-reduced Matrigel (BD Biosciences) was thawed at 4°C. After the Matrigel changed to a liquid state, 50 μl per well was added to a 96-well plate for polymerization. Human umbilical vein endothelial cells (HUVECs) (2 × 10^4^/well) were seeded on top of the Matrigel and cultured with the supernatants from HepG2 or steatotic HepG2 cells for 24 h. Meanwhile, we set a program on the IncuCyte ZOOM to record an image every hour. The cumulative number of tubes in every well in 5 random microscopic fields was calculated by ImageJ software. The experiments were performed at least in triplicate.

### Statistical analysis

Statistical data were analysed by SPSS version 13.0 (Chicago, IL. USA). The correlations between HIF-2α levels and clinical characteristics were evaluated using the Pearson Chi-squared test. Overall survival (OS) curves were plotted by Kaplan-Meier survival analysis and log-rank test. Moreover, predictive variables, such as tumour size, were estimated by univariate and multivariate analyses. Student’s t-test and ANOVA were used to evaluate the differences among groups. We considered differences statistically significant when the p values were less than 0.05.

## CONCLUSIONS

In conclusion, in a hypoxic microenvironment, HIF-2α upregulation promotes steatotic HCC progression by activating lipid synthesis via the PI3K-AKT-mTOR pathway. Therefore, HIF-2α may be a target for specific therapeutic measures for NAFLD-HCC patients.

## Supplementary Material

Supplementary Figure 1

Supplementary Table 1
