# Supplementary material for: HIF-2α upregulation mediated by hypoxia promotes NAFLD-HCC progression by activating lipid synthesis via the PI3K-AKT-mTOR pathway
Source: Aging (Albany NY). 2019 Dec 4;11(23):10839–60. doi: 10.18632/aging.102488 (PMC6932893; doi:10.18632/aging.102488)
Supplement: Supplementary Figure 1 [file aging-11-102488-s002..pdf]

SUPPLEMENTARY FIGURE

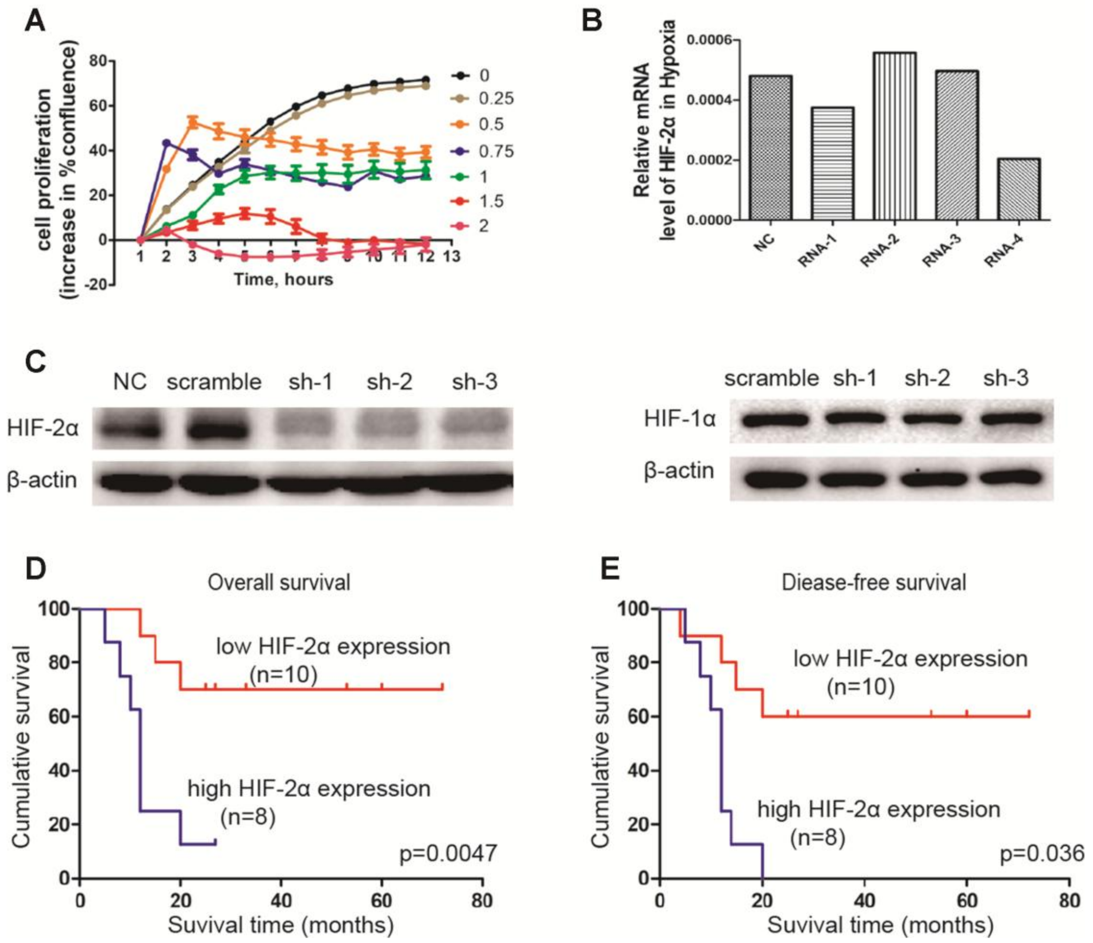

Supplementary Figure 1. (**A**), Summary graph showing the HCC cell 2D growth rate in different FFA concentration. (**B**), Quantitative RT-PCR assessment of HIF-2α expression in sh-HIF2α HCC cells under hypoxic conditions. Transcription levels were normalized to those of β-actin. (**C**) Western blot analysis of HIF-2α and HIF-1α expression in sh-HIF2α HCC cells under hypoxic conditions. β-actin was used as the loading control. (**D**,**E**) Survival curves of 18 NAFLD-HCC patients stratified according to HIF-2α protein expression.
